# Role of CRISPR-Cas system on antibiotic resistance patterns of *Enterococcus faecalis*

**DOI:** 10.1186/s12941-021-00455-6

**Published:** 2021-07-28

**Authors:** Pourya Gholizadeh, Mohammad Aghazadeh, Reza Ghotaslou, Mohammad Ahangarzadeh Rezaee, Tahereh Pirzadeh, Longzhu Cui, Shinya Watanabe, Hadi Feizi, Hiva Kadkhoda, Hossein Samadi Kafil

**Affiliations:** 1 Drug Applied Research Center, Tabriz University of Medical Sciences, Tabriz, Iran; 2 Student Research Committee, Tabriz University of Medical Sciences, Tabriz, Iran; 3 Immunology Research Center, Tabriz University of Medical Sciences, Tabriz, Iran; 4 Division of Bacteriology, Department of Infection and Immunity, School of Medicine, Jichi Medical University, Shimotsuke, Tochigi Japan

**Keywords:** *Enterococcus faecalis*, CRISPR-cas system, Antibiotic resistance, Multi-drug resistance

## Abstract

Clustered regularly interspaced short palindromic repeat (CRISPR)-Cas systems are one of the factors which can contribute to limiting the development and evolution of antibiotic resistance in bacteria. There are three genomic loci of CRISPR-Cas in *Enterococcus faecalis*. In this study, we aimed to assess correlation of the CRISPR-Cas system distribution with the acquisition of antibiotic resistance among *E. faecalis* isolates. A total of 151 isolates of *E. faecalis* were collected from urinary tract infections (UTI) and dental-root canal (DRC). All isolates were screened for phenotypic antibiotic resistance. In addition, antibiotic resistance genes and CRISPR loci were screened by using polymerase chain reaction. Genomic background of the isolates was identified by random amplified polymorphic DNA (RAPD)-PCR. The number of multidrug-resistant *E. faecalis* strains were higher in UTI isolates than in DRC isolates. RAPD-PCR confirmed that genomic background was diverse in UTI and DRC isolates used in this study. CRISPR loci were highly accumulated in gentamycin-, teicoplanin-, erythromycin-, and tetracycline-susceptible strains. In concordance with drug susceptibility, smaller number of CRISPR loci were identified in *vanA*, *tetM*, *ermB*, *aac6’-aph(2”)*, *aadE*, and *ant(6)* positive strains. These data indicate a negative correlation between CRISPR-*cas* loci and antibiotic resistance, as well as, carriage of antibiotic resistant genes in both of UTI and DRC isolates.

**Supplementary Information:**

The online version contains supplementary material available at 10.1186/s12941-021-00455-6.

## Introduction

Over the past decades, commensal bacteria and opportunistic pathogens including *Enterococcus* spp. have been considered as serious public health threats, and the therapeutic options have become limited. *Enterococcus* spp. are gram-positive, facultative anaerobes, catalase-negative cocci, which are found in a variety environments such as nature, water, soil, food, avian, mammalian, and human gastrointestinal tracts [1]. The important species within the genus *Enterococcus* are *E. faecalis* and *E. faecium*, which have been reported to be opportunistic pathogens for up to 90 % of human enterococcal infections [2]. Clinical studies demonstrated that *E. faecalis* are frequently isolated from community- and nosocomial-acquired infections such as bacteremia, urinary tract infections (UTIs), endocarditis and soft tissue infections [2, 3], as well as from untreated and previously treated root canals infections [4, 5]. Antibiotic resistance in enterococci is a challenge in the clinical setting, and reduces the efficacy of treatment of Enterococcal infectious diseases [6]. *E. faecalis* expresses an intrinsic resistance to several antibiotic groups and biocides, including beta-lactams, glycopeptides, fluoroquinolones, as well as high-level resistance to aminoglycosides including gentamicin and streptomycin [7]. In addition, enterococci can transfer antibiotic resistance to other bacteria through mobile genetic elements such as transposons and plasmids [6]. Owing to highly efficient mechanisms of enterococci for the distribution and acquisition of antibiotic resistance genes, as well as, high frequency of the transfer and exchange of resistance genes between resistant strains and virulent strains, enterococci are considered as reservoirs of antibacterial resistance genes [8]. In addition, they are important indicators of antibiotic resistance and can help in tracking the evolution of antibiotic resistance in different environments [8, 9].

One of the factors that could limit the development and evolution of antibiotic resistance in bacteria is clustered regularly interspaced short palindromic repeat (CRISPR)-Cas systems [10–14]. CRISPR-Cas systems are widespread among archaea and bacteria, which protect these organisms against mobile genetic elements such as phages, plasmids and transposons [10, 13–15]. The mechanism of action of these systems are included in three steps of adaptation, expression and interference [16]. Genome analysis suggested that CRISPR-Cas systems interact with mobile elements. *E. faecalis* has a single type of CRISPR-Cas, type II. There are three loci of CRISPR in the genomes of *E. faecalis*, which are including CRISPR1-Cas, CRISPR2 and CRISPR3-Cas [12, 17, 18]. CRISPR1-Cas were first found in the *E. faecalis* OG1RF strain, and is identified between the V583 homologues of open reading frames (ORFs) EF0672 and EF0673. CRISPR2 is an orphan CRISPR, consisting only of palindromes and spacers without any *cas* genes, and is identified between the V583 homologues of ORFs EF2062 and EF2063 [17]. CRISPR3-Cas was found in the genomes of *E. faecalis* strains Fly1 and T11 that is identified between the homologues of the *E. faecalis* V583 ORFs EF1760 and EF1759 [12]. Nmeni subtype loci marker gene *cas1* and *cas2*, as well as, Nmeni subtype-specific genes *csn1* and *csn2* were found in both CRISPR1-Cas and CRISPR3-Cas [12, 17]. Several studies have demonstrated that the CRISPR-Cas system has applications for genome engineering and exerts a strong selective pressure for the acquisition of antibiotic resistance and virulence factors in bacteria [10–12, 19]. Therefore, in this study, we aimed to assess correlation of the CRISPR-Cas system distribution on the acquisition of antibiotic resistance among *E. faecalis* isolates.

## Methods and materials

### Bacterial strains

This study was approved by the Regional Ethics Committee of Tabriz (Tabriz University of Medical Sciences, Tabriz, Iran, No. IR.TBZMED.REC.1397.188). A total of 144 isolates of *E. faecalis* were collected: 75 isolates were from urinary tract infections (UTIs) and 69 isolates were from dental-root canal (DRC) infections. The UTI isolates collected from the patients admitted to Imam Reza Teaching and Treatment Hospital and pediatric hospitals of Tabriz, Iran. The DRC isolates were collected from patients referred to the clinic of the Faculty of Dentistry at Tabriz University of Medical Sciences, Tabriz, Iran, for treatment. The DRC isolates were collected using procedure as described by Gomes et al. [20]. The paper points were transferred to a tube containing Enterococcal broth (Becton Dickenson microbiology systems, Cockeysville, MD) and cultured on a bile esculin azide agar (Himedia, India) and incubated at 37 ºC for 24–48 h [20]. Suspected colonies were identified by the standard procedures of microbiology [21, 22] and genotype detection was performed by *ddlE* primer [23, 24], as shown in Additional file 1: Table S1. Both clinical and DRC isolates were stored in a trypticase soy broth containing 10 % glycerol at −70 ºC for further studies.

### Phenotypic antibiotic susceptibility assay

Disk diffusion procedure on Muller-Hinton agar medium (Merck, Germany) was performed for all *E. faecalis* isolates according to the clinical and laboratory standard institute (CLSI) guidelines [25]. Eleven antibiotics tested were included penicillin (10U), ampicillin (10 µg), gentamicin (120 µg), vancomycin (30 µg), teicoplanin (30 µg), erythromycin (15 µg), tetracycline (30 µg), ciprofloxacin (5 µg), rifampin (5 µg), fosfomycin (200 µg) and linezolid (30 µg) (Mast, UK).

In addition, minimum inhibitory concentration (MIC) of vancomycin (CAS: 1404-93-9, Sigma-Aldrich, USA) and gentamicin (CAS: 1405-41-0, Sigma-Aldrich, USA) were determined using the agar dilution procedure as recommended by CLSI [25]. Following disk diffusion and agar dilution procedures, plates were incubated at 35 ºC for 18–24 h. The MIC50 value was defined as the concentration that inhibited at least 50 % of the isolates and MIC90 was defined as the concentration that inhibited at least 90 % of the isolates.

### Genotypic detection of antibiotic resistance genes

Total DNA of all *E. faecalis* isolates were extracted using tissue buffer procedure (0.25 % sodium dodecyl sulfate (SDS) and 0.05 M NaOH). Genotypic analysis for isolates that displayed resistance to antibiotics phenotypically was accomplished for the presence of antibacterial resistance genes using polymerase chain reaction (PCR). Genes for resistance to penicillin (*blaZ*), macrolide (*ermA* and *ermB*), tetracycline (*tetM* and *tetO*), vancomycin (*vanA* and *vanB*), and aminoglycoside (*aac6’-aph(2”)*, *aadE* and *ant(6)*) were tested among the isolates. Primers used for detection of the antibiotic resistance genes are shown in Additional file 1: Table S1. PCR amplification were performed in a 25 µl reaction mixture using 2 µl of template DNA, 2 mM MgCl2, 1 µM of each primer and 1U of Taq DNA polymerase (Yekta Tajhiz Azma, Iran). The PCR products were analyzed by electrophoresis using a 1 % agarose gel in 1X TBE buffer and the stained gels were viewed using a standard UV transilluminator. *E. faecalis* ATCC®29,212™ and E. *faecalis* MMH594, *E. faecium* 15555EK (positive for *tet*M, *erm*B), *E. faecalis* 16680EK (positive for *aac(6’)-aph(2”); ant(6)*), *E. faecalis* E206 (positive for *vanA*), *E. faecalis* E2781 (positive for *vanB*) were used as control strains.

### Detection of CRISPR-Cas loci

The presence of CRISPR loci were identified by five primer sets including three CRISPR loci and *cas* genes of CRISPR1 and CRISPR3 (see Additional file 1: Table S1). The mix for each loci contained 25 µl of the PCR master mix (Yekta Tajhiz Azma, Iran), 2 µl of template DNA and 1 µM of each primer. The amplification condition was carried out with the following thermal cycling conditions: an initial denaturation at 94 ºC for 5 min, 30 cycles of denaturation (94 ºC for 30 s), annealing (60 ºC for 30 s), and elongation (72 ºC for 45 s), followed by final elongation at 72 ºC for 5 min. The PCR products were analyzed by electrophoresis using a 1 % agarose gel in 1X TBE buffer and the stained gels were viewed using a standard UV transilluminator.

### Analysis of genotype by RAPD-PCR

All *E. faecalis* isolates were genotyped by the single primer M13 (5’-GAGGGTGGCGGTTCT-3’) [26]. Reactions were carried out in a total volume of 25 µl containing 2 µl of template DNA, 3 mM of MgCl2, 1 µM of M13 primer and 1 U of Taq DNA polymerase. The PCR cycling program consisted of an initial denaturation at 94 ºC for 5 min, 30 cycles of 95 ºC for 60 s, 42 ºC for 30 s (with 0.6 ºC/s ramp), and 72 ºC for 60 s and a final elongation at 72 ºC for 5 min. All PCR products were separated by electrophoresis on 1.5 % agarose gel in 1X TBE buffer and stained by ethidium bromide. All generated RAPD-PCR fingerprints were analyzed by GelJ v.2 software. The similarity matrix of the generated fingerprints was based on the Pearson product-moment correlation coefficient. A cluster analysis was deduced using the unweighted-pair-group method with arithmetic averages (UPGMA).

### Statistical analysis

SPSS software, version 20.0, (Chicago, IL, USA) was used for statistical analysis. One-tailed Fisher’s exact test was used to compare the occurrence of different antibiotic resistance, genes and CRISPR-*cas* loci among UTIs and DRC isolates. In addition, Spearman’s rank correlation coefficient was calculated between the presence of different antibiotic resistance, genes and CRISPR-*cas* loci among isolates. Mann-Whitney U test was used for comparison of the numbers of antibiotic resistance and related genes between isolates with and without CRISPR loci. Significance was set at p-value < 0.05.

## Results

A total of 144 isolates of *E. faecalis* were included in the study, 75 isolates were from urinary tract infections (UTIs) and 69 isolates were from dental-root canal (DRC) infections. The UTI specimens were obtained from different wards including outpatients (32, 42.67 %), internal medicine ward (18, 24 %), intensive-care units (ICU) (12, 16 %), infectious ward (8, 10.67 %), emergency ward (3, 4 %), urology and nephrology (1, 1.33 %) and ear-nose-throat (ENT) (1, 1.33 %). The age range of the patients with UTIs was from 5 months to 86 years, with a mean of 37.5 (± 31.69) years. In the UTI isolates, 37 (49.33 %) isolates were obtained from female and 38 (50.67 %) isolates were from male patients. 45 (65.22 %) of the DRC isolates were obtained from the males and 24 (34.78 %) from the females. The age range of the endodontic treatment patients was 12–66 years, with a mean of 32.53 (± 10.84) years. All isolates were investigated for the antibiotic susceptibility assay and phenotypic characteristics of antibiotic resistance among UTI and DRC isolates are shown in Table 1. Overall, the occurrence of penicillin, erythromycin, tetracycline, ciprofloxacin, teicoplanin and gentamicin resistance were present in proportionally higher numbers of UTI isolates than DRC isolates (p < 0.05). The results of MIC demonstrated that nine (5.96 %) isolates were resistant to vancomycin of which MIC50 and MIC90 were 2 µg/mL and 4 µg/mL, respectively. As well as, 49 (34.03 %) isolates showed MIC values greater than 128 µg/mL against gentamicin (MIC50 < 16 µg/mL and MIC90 > 512 µg/mL). The MIC values of vancomycin and gentamicin of UTI and DRC isolates are shown in Table 2. In addition, the isolates resistant to more than two antibiotics were considered as multidrug resistance (MDR). Antibiotic patterns of MDR UTI and DRC isolates of *E. faecalis* are shown in Table 3. Overall, 59 types of antibiotic resistance patterns existed in this study, of which 44 types were MDR and there was four (2.78 %) isolates resistant to nine antibiotics, this was the highest antibiotic resistance observed. 80 out of 151 (55.56 %) isolates were considered as MDR, among which 63 (78.75 %) were UTI isolates and 17 (21.25 %) were DRC isolates. The antibiotic resistance counts in UTI isolates were significantly higher in comparison to DRC isolates (p < 0.001). Antibiotic patterns of MDR UTIs and DRC isolates of *E. faecalis* are shown in Table 3.

**Table. 1:** Phenotypic characteristics of UTIs and DRC isolates of *E. faecalis*

Antibiotics	Total resistant isolates (%)	UTI resistant isolates (%)	DRC resistant isolates (%)	p-value
Ampicillin	10 (6.94 %)	7 (9.33 %)	3 (4.34 %)	0.199
Penicillin	33 (22.92 %)	30 (40.00 %)	3 (4.35 %)	< 0.001
Vancomycin	10 (6.94 %)	8 (10.67 %)	2 (2.90 %)	0.064
Teicoplanin	11 (7.64 %)	9 (12.00 %)	2 (2.90 %)	0.038
Fosfomycin	28 (19.44 %)	17 (22.67 %)	11 (15.94 %)	0.210
Erythromycin	70 (48.61 %)	62 (82.67 %)	8 (11.60 %)	< 0.001
Linezolid	2 (1.38 %)	2 (2.67 %)	0	0.270
Tetracycline	87 (60.42 %)	68 (90.67 %)	19 (27.54 %)	< 0.001
Ciprofloxacin	65 (45.14 %)	49 (65.33 %)	16 (23.20 %)	< 0.001
Gentamicin (128 µg)	49 (34.03 %)	41 (54.67 %)	8 (11.60 %)	< 0.001
Rifampin	99 (68.75 %)	49 (65.33 %)	50 (72.47 %)	0.229

p-value was calculated by one-tailed Fisher’s exact test and p-value < 0.05 was significant

**Table. 2:** MIC values of vancomycin and gentamicin among UTIs and DRC isolates of *E. faecalis*

MIC of vancomycin	Total isolates (%)	UTI isolates (%)	DRC isolates (%)	MIC of gentamicin	Total isolates (%)	UTI isolates (%)	DRC isolates (%)
1≥	70 (48.61 %)	35 (46.67 %)	35 (50.72 %)	16>	91 (63.19 %)	33 (44.00 %)	58 (84.06 %)
2	55 (38.19 %)	24 (32.00 %)	31 (44.93 %)	64	4 (2.78 %)	1 (1.33 %)	3 (4.35 %)
4	9 (6.25 %)	9 (10.67 %)	1 (1.45 %)	256	0	0	0
8	1 (0.69 %)	0	1 (1.45 %)	512	6 (4.17 %)	6 (8.00 %)	0
256	2 (1.39 %)	1 (1.33 %)	1 (1.45 %)	512<	43 (29.86 %)	35 (46.67 %)	8 (11.59 %)
512<	7 (4.86 %)	7 (9.33 %)	0	–	–	–	–

**Table. 3:** Antibiotic patterns of MDR UTIs and DRC isolates of *E. faecalis*

Antibiotics to which isolates showed resistance	No. of antibiotic	Total isolates (%)	UTI isolates (%)	DRC isolates (%)
F, Tt, R	3	2 (1.39 %)	1 (1.33 %)	1 (1.45 %)
E, Tt, R	3	4 (2.78 %)	4 (5.33 %)	0
E, T, G	3	1 (0.69 %)	1 (1.33 %)	0
Tt, C, R	3	4 (2.78 %)	3 (4.00 %)	1 (1.45 %)
E, C, R	3	1 (0.69 %)	1 (1.33 %)	0
E, Tt, G	3	1 (0.69 %)	1 (1.33 %)	0
E, Tt, C	3	1 (0.69 %)	1 (1.33 %)	0
E, P, Tt	3	1 (0.69 %)	1 (1.33 %)	0
Tt, G, R	3	1 (0.69 %)	0	1 (1.45 %)
E, G, R	3	5 (3.47 %)	0	5 (7.25 %)
A, P, Tt	3	1 (0.69 %)	0	1 (1.45 %)
A, P, C	3	1 (0.69 %)	0	1 (1.45 %)
F, Tt, C	3	1 (0.69 %)	0	1 (1.45 %)
F, P, Tt, R	4	1 (0.69 %)	1 (1.33 %)	0
E, Tt, C, R	4	1 (0.69 %)	1 (1.33 %)	0
E, Tt, C, G	4	4 (2.78 %)	4 (5.33 %)	0
E, P, Tt, R	4	2 (1.39 %)	2 (2.67 %)	0
E, Tt, G, R	4	2 (1.39 %)	2 (2.67 %)	0
E, L, Tt, R	4	1 (0.69 %)	1 (1.33 %)	0
P, Tt, C, G	4	1 (0.69 %)	1 (1.33 %)	0
F, Tt, T, R	4	2 (1.39 %)	0	2 (2.90 %)
E, C, G, R	4	1 (0.69 %)	0	1 (1.45 %)
F, Tt, C, R	4	1 (0.69 %)	0	1 (1.45 %)
E, Tt, C, G, R	5	7 (4.86 %)	7 (9.33 %)	0
E, A, P, Tt, C	5	1 (0.69 %)	1 (1.33 %)	0
E, P, Tt, C, R	5	1 (0.69 %)	1 (1.33 %)	0
E, P, Tt, G, R	5	1 (0.69 %)	1 (1.33 %)	0
E, P, Tt, C, G	5	3 (2.08 %)	3 (4.00 %)	0
F, E, A, P, Tt	5	1 (0.69 %)	0	1 (1.45 %)
F, E, Tt, C, R	5	1 (0.69 %)	0	1 (1.45 %)
E, L, Tt, C, G, R	6	1 (0.69 %)	1 (1.33 %)	0
F, E, Tt, C, G, R	6	4 (2.78 %)	4 (5.33 %)	0
E, P, Tt, C, G, R	6	7 (4.86 %)	7 (9.33 %)	0
V, E, Tt, C, G, T	6	1 (0.69 %)	1 (1.33 %)	0
F, E, P, Tt, C, G, R	7	1 (0.69 %)	1 (1.33 %)	0
F, E, A, P, Tt, C, G	7	1 (0.69 %)	1 (1.33 %)	0
V, F, E, P, Tt, C, T	7	1 (0.69 %)	1 (1.33 %)	0
E, A, P, Tt, C, G, R	7	1 (0.69 %)	1 (1.33 %)	0
F, E, P, Tt, C, G, T, R	8	1 (0.69 %)	1 (1.33 %)	0
V, F, E, A, P, C, G, T	8	1 (0.69 %)	1 (1.33 %)	0
F, E, A, P, Tt, C, G, R	8	1 (0.69 %)	1 (1.33 %)	0
V, F, E, P, Tt, C, G, T	8	1 (0.69 %)	1 (1.33 %)	0
V, F, E, P, Tt, C, G, T, R	9	2 (1.39 %)	2 (2.67 %)	0
V, F, E, A, P, T, C, T, R	9	1 (0.69 %)	1 (1.33 %)	0
V, F, E, A, P, C, G, T, R	9	1 (0.69 %)	1 (1.33 %)	0
Total	-	80 (55.55 %)	63 (78.75 %)	17 (21.25 %)

*V *Vancomycin, *F *Fosfomycin, *E *Erythromycin, *A *Ampicillin, *L *Linezolid, *P *Penicillin, *Tt *Tetracycline, *C *Ciprofloxacin, *G *Gentamicin, *T *Teicoplanin, *R *Rifampin

**Fig. 1:**
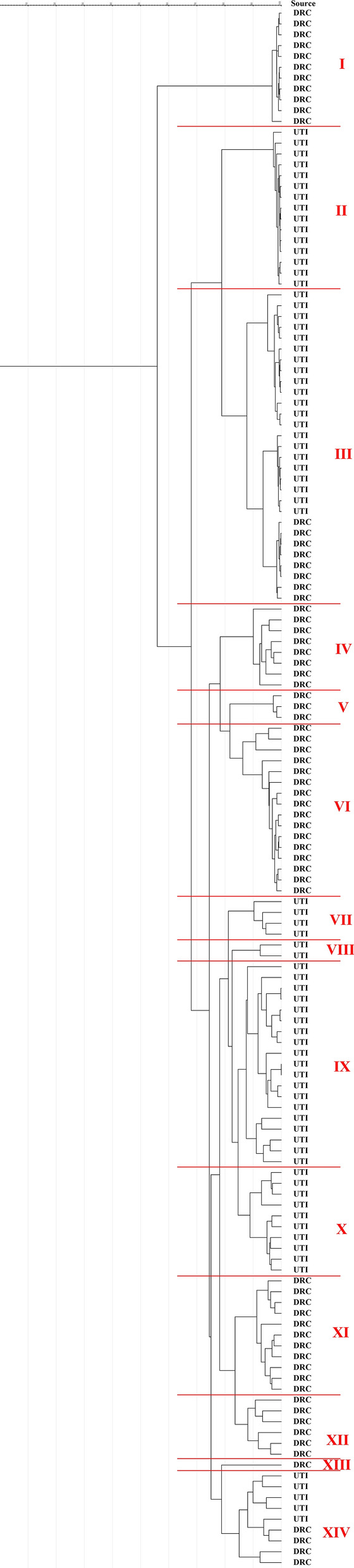
Cluster analysis of genetic fingerprints of UTIs and DRC isolates of *E. faecalis* by the use of RAPD-PCR. The similarity matrix of the generated fingerprints was based on the Pearson product-moment correlation coefficient. A cluster analysis was deduced using the unweighted-pair-group method with arithmetic averages (UPGMA). The isolates differentiation was achieved at a cut off level of 85 %. Overall, fourteen clusters were included I-XIV, which each cluster contained 4–19 isolates exception of cluster VIII and XIII that cluster XIII had 1 isolate of the DRC and cluster VIII had 2 isolates of the UTIs

The banding patterns, analyzed by GelJ software, showed ten major groups of all UTI and DRC isolates at a cut off level of 85 % (Fig. 1). Overall, 14 clusters were obtained (cluster I-XIV), which each cluster contained 1–19 isolates. The UTI isolates were distributed in clusters II, III, VII, VIII, IX, X, and XIV while DRC isolates were distributed in clusters I, III, IV, V, VI, XI, XII, XIII and XIV. Therefore, clusters II, VII, VIII, IX and X contained only UTI isolates and clusters I, IV, V, VI, XI, XII and XIII contained only DRC isolates. Distribution of CRISPR loci among RAPD clusters of the isolates are shown in Table 4. There were six clusters with isolates containing CRISPR1 and eight clusters without it, which the most frequent of isolates with CRISPR1 were found in clusters II (4 isolates) and III (5 isolates). The most frequent of isolates with CRISPR2 were found in clusters III (15 isolates) and IX (13 isolates), while it was not found in cluster I. As well as, there were 7 clusters with isolates that without CRISPR3 and the most frequent clusters were III (8 isolates) and XII (5 isolates).

**Table. 4:** Distribution of CRISPR loci among RAPD clusters of *E. faecalis* isolates

RAPD types	CRISPR1-Present	CRISPR1-Absent	CRISPR2-Present	CRISPR2-Absent	CRISPR3-Present	CRISPR3-Absent
I	0	11	0	11	3	8
II	4	11	4	11	0	15
III	5	24	15	14	8	21
IV	0	8	5	3	2	6
V	1	7	7	1	0	8
VI	2	9	3	8	1	10
VII	0	4	1	3	0	4
VIII	1	1	2	0	0	2
IX	2	17	13	6	0	19
X	0	10	5	5	0	10
XI	0	11	11	0	4	7
XII	0	6	4	2	5	1
XIII	0	1	1	0	0	1
XIV	0	9	6	3	4	5

The occurrence of antibacterial resistance genes in *E. faecalis* isolates of UTI and DRC isolates is shown in Table 5. Overall, *aadE* (69.44 %) and *tetM* (63.89 %) genes were present in most samples, while *ermA* (3.47 %) and *tetO* (4.17 %) genes were present in least of them; none of the samples were found to possess *vanB*. The occurrence of *tetM*, *ermA*, *ermB*, *vanA*, *aac6’-aph(2”)*, *aadE* and *ant(6)* in UTI isolates were significantly predominant in comparison to DRC isolates (p < 0.05). In addition, the resistance genes counts in UTI isolates were significantly predominant in comparison to DRC isolates (p < 0.001). As well as, the results demonstrated that the resistance genes counts were directly associated with antibiotic resistance counts (p < 0.001). The antibiotic resistance gene patterns among UTIs and DRC isolates of *E. faecalis* are shown in Table 6.

**Table. 5:** Occurrence of antibiotic resistance-related genes among UTIs and DRC isolates of *E. faecalis*

Gene	Total isolates (%)	UTI isolates (%)	DRC isolates (%)	p-value
*blaZ*	8 (5.56 %)	2 (2.67 %)	6 (8.70 %)	0.112
*tetM*	92 (63.89 %)	71 (94.67 %)	21 (30.43 %)	< 0.001
*tetO*	6 (4.17 %)	5 (6.67 %)	1 (1.45 %)	0.125
*ermA*	5 (3.47 %)	5 (6.67 %)	0	0.036
*ermB*	59 (40.97 %)	52 (69.33 %)	7 (10.14 %)	< 0.001
*vanA*	9 (6.25 %)	8 (10.67 %)	1 (1.45 %)	0.023
*vanB*	0	0	0	–
*aac6’-aph(2”)*	49 (34.03 %)	43 (57.33 %)	6 (8.70 %)	< 0.001
*aadE*	100 (69.44 %)	64 (85.33 %)	36 (52.17 %)	< 0.001
*ant(6)*	50 (34.72 %)	47 (62.67 %)	3 (4.35 %)	< 0.001

p-value was calculated by one-tailed Fisher’s exact test and p-value < 0.05 was significant

**Table. 6:** Antibiotic resistance gene patterns of UTIs and DRC isolates of *E. faecalis*

Resistance gene profiles	No. of genes	Total (%)	UTI isolates (%)	DRC isolates (%)
0	0	23 (15.97 %)	0	23 (33.33 %)
tM	1	10 (6.94 %)	4 (5.33 %)	6 (8.70 %)
aE	1	14 (9.72 %)	0	14 (20.29 %)
bZ	1	1 (0.69 %)	0	1 (1.45 %)
tM, eA	2	1 (0.69 %)	1 (1.33 %)	0
tM, aE	2	16 (11.11 %)	7 (9.33 %)	9 (13.04 %)
tM, eB	2	3 (2.08 %)	2 (2.67 %)	1 (1.45 %)
tO, aE	2	2 (1.39 %)	1 (1.33 %)	1 (1.45 %)
tM, at6	2	1 (0.69 %)	1 (1.33 %)	0
a6, aE	2	1 (0.69 %)	0	1 (1.45 %)
bZ, aE	2	4 (2.78 %)	0	4 (5.80 %)
bZ, tM	2	1 (0.69 %)	0	1 (1.45 %)
tM, vA	2	1 (0.69 %)	0	1 (1.45 %)
tM, eA, aE	3	1 (0.69 %)	1 (1.33 %)	0
tM, a6, aE	3	5 (3.47 %)	4 (5.33 %)	1 (1.45 %)
tM, tO, aE	3	1 (0.69 %)	1 (1.33 %)	0
tM, eB, aE	3	5 (3.47 %)	3 (4.00 %)	2 (2.90 %)
tM, tO, eB	3	1 (0.69 %)	1 (1.33 %)	0
eB, a6, aE	3	1 (0.69 %)	0	1 (1.45 %)
tM, eA, a6, aE	4	1 (0.69 %)	1 (1.33 %)	0
tM, eA, eB, a6	4	1 (0.69 %)	1 (1.33 %)	0
tM, eB, aE, at6	4	8 (5.56 %)	8 (10.67 %)	0
tM, eB, a6, aE	4	1 (0.69 %)	1 (1.33 %)	0
eB, a6, aE, at6	4	4 (2.78 %)	1 (1.33 %)	3 (4.35 %)
tM, eB, a6, at6	4	1 (0.69 %)	1 (1.33 %)	0
tM, eB, a6, aE, at6	5	25 (17.36 %)	25 (33.33 %)	0
bZ, tM, a6, aE, at6	5	1 (0.69 %)	1 (1.33 %)	0
eB, vA, a6, aE, at6	5	2 (1.39 %)	2 (2.67 %)	0
tM, eB, vA, aE, at6	5	1 (0.69 %)	1 (1.33 %)	0
bZ, tM, vA, aE, at6	5	1 (0.69 %)	1 (1.33 %)	0
tM, tO, eB, a6, aE, at6	6	2 (1.39 %)	2 (2.67 %)	0
tM, eB, vA, a6, aE, at6	6	3 (2.08 %)	3 (4.00 %)	0
tM, eA, eB, vA, a6, aE, at6	7	1 (0.69 %)	1 (1.33 %)	0
Total	-	144 (100 %)	80 (100 %)	69 (100 %)

bZ: *blaZ*; tM: *tetM*; tO: *tetO*; eA: *ermA*; eB: *ermB*; vA: *vanA*; a6: *aac(6’)-aph(2”)*; aE: *aadE*; at6: *ant(6)*

**Fig. 2:**
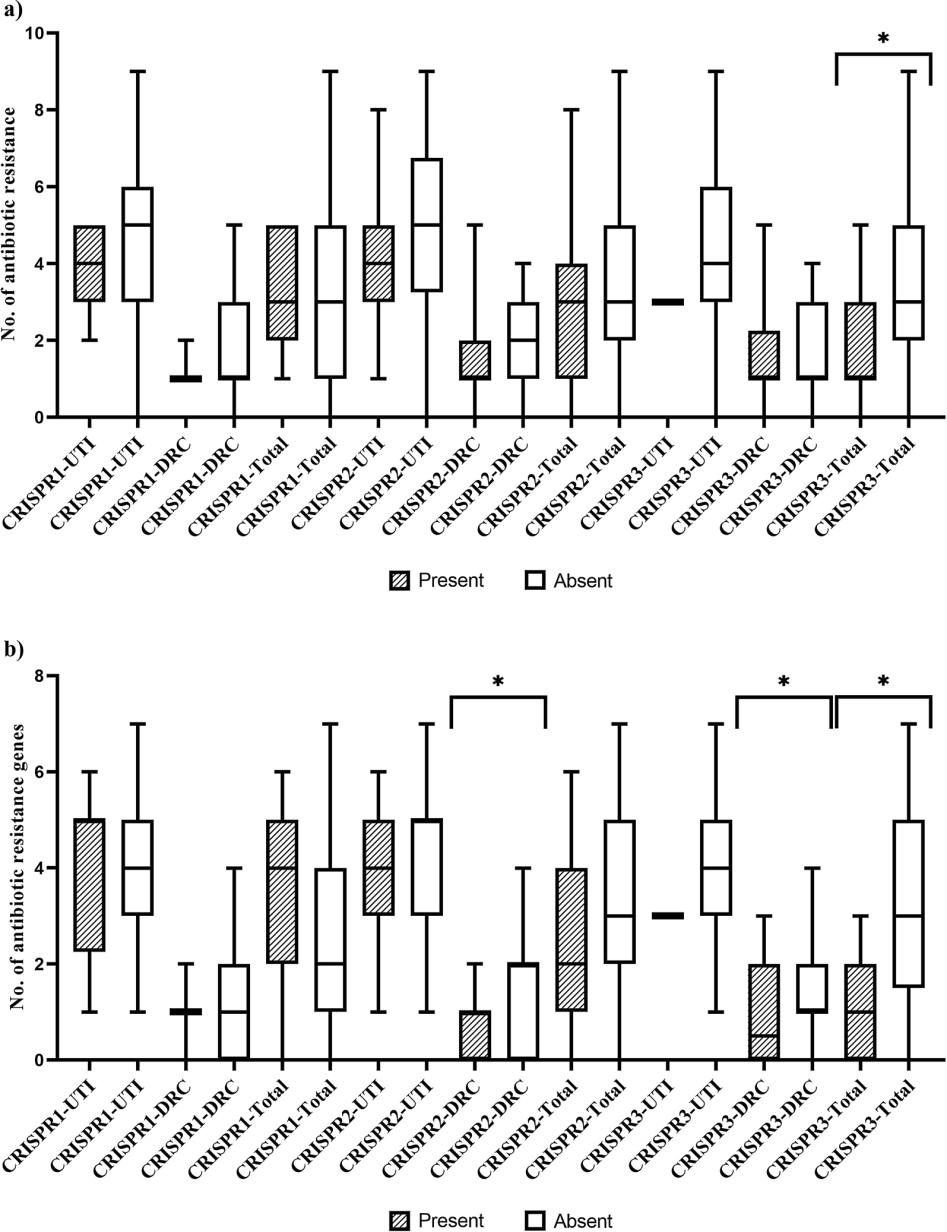
Correlation between the Number of resistant antibiotics (a) and antibiotic resistance genes (b) in each *E. faecalis* isolate and occurrence of CRISPR loci. Note: *: p-value was significant (p-value < 0.05), which was calculated by U Mann-Whitney test

**Fig. 3:**
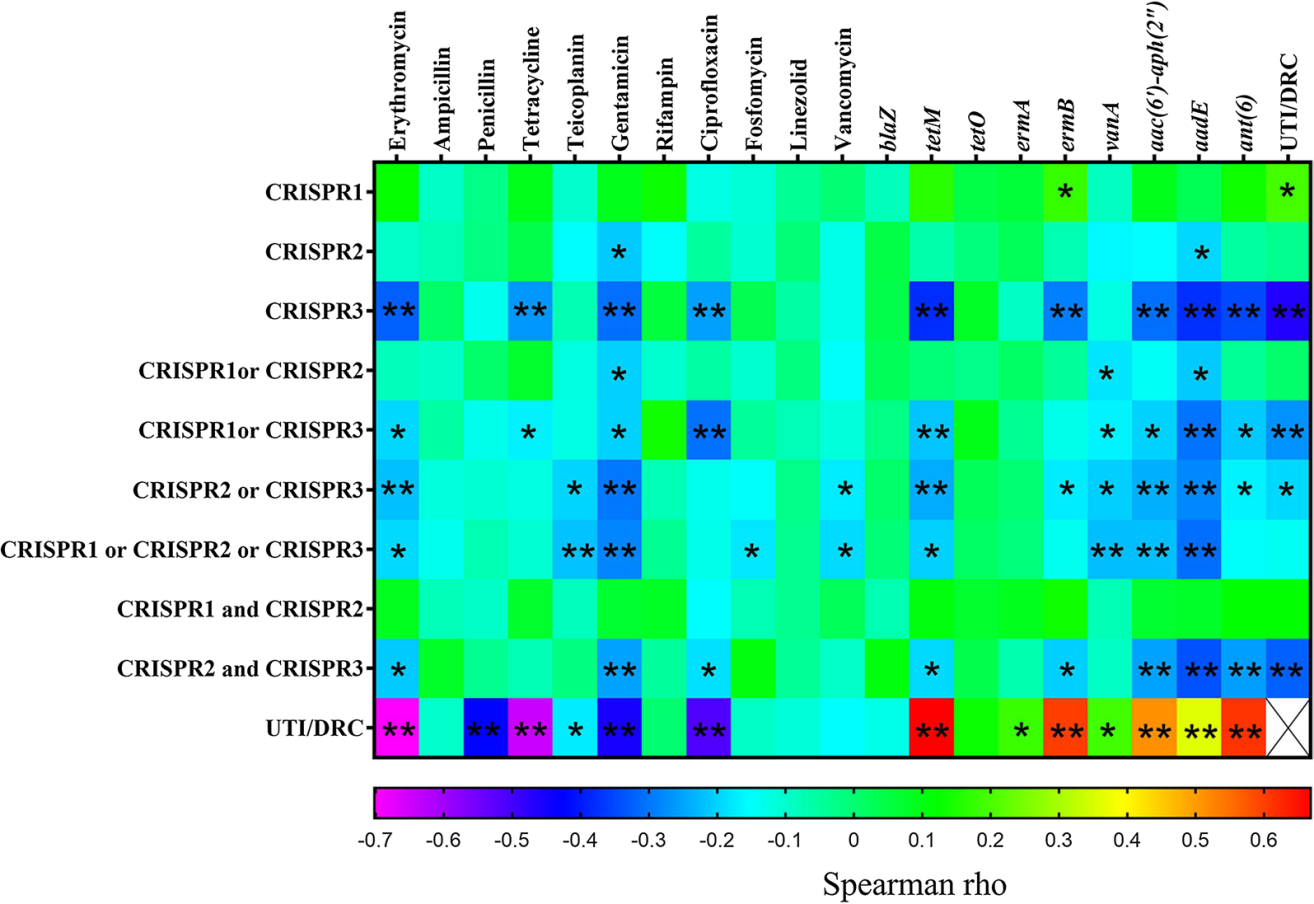
Correlation between antibiotic resistance, genes and CRISPR loci among *E. faecalis* isolates (– 0.380 < Spearman rho < 0.178). Note: *: Correlation is significant at the 0.05 level; **: Correlation is significant at the 0.01 level. Correlations were calculated by Spearman’s rank correlation coefficient

The occurrence of CRISPR-*cas* in *E. faecalis* isolates of UTI and DRC isolates is shown in Table 7. Overall, CRISPR2 was identified in 77 (53.47 %) of the isolates, followed by CRISPR3 and CRISPR1 (17.75 and 10.42 %, respectively). The correlation between antibiotic resistance and antibiotic resistance genes counts and the occurrence of CRISPR loci are shown in Fig. 2. The presence of CRISPR2 and CRISPR3 were indirectly associated to the counts of resistant antibiotics and related genes among the isolates (p < 0.05). The correlation between the presence of CRISPR-Cas and phenotypic antibiotic resistance were shown in Fig. 3. Overall, CRISPR2 was predominant in gentamicin and rifampin susceptible isolates (p = 0.009 and p = 0.054, respectively). In addition, CRISPR3 was predominant in erythromycin, tetracycline, ciprofloxacin and gentamicin susceptible isolates (p < 0.001, p = 0.002, p = 0.002 and p < 0.001, respectively). No correlation was found between CRISPR1 and antibiotic resistance. The absence of CRISPR3 was significantly associated with the increased values of vancomycin MIC (p < 0.001). As well as, the absence of CRISPR2 and CRISPR3 were significantly associated to the increased values of gentamicin MIC (p = 0.038 and p < 0.001, respectively). In UTI isolates, CRISPR2 was predominant in fosfomycin, ampicillin, and teicoplanin susceptible isolates (p = 0.032, p = 0.042, and p = 0.059, respectively). In addition, CRISPR2 was predominant in tetracycline resistance isolates (p = 0.004). In DRC isolates, CRISPR2 was predominant in rifampin and gentamicin susceptible isolates (p = 0.049 and p = 0.001, respectively).

**Table. 7:** Occurrence of CRISPR-*cas* among UTIs and DRC isolates of *E. faecalis*

CRISPR locus	Total (%)	UTI isolates (%)	DRC isolates (%)	p-value
CRISPR1	15 (10.42 %)	12 (16.00 %)	3 (4.35 %)	0.020
CRISPR2	77 (53.47 %)	39 (52.00 %)	38 (55.07 %)	0.420
CRISPR3	27 (17.75 %)	1 (1.33 %)	26 (37.68 %)	< 0.001
CRISPR1 + CRISPR2	11 (7.64 %)	8 (10.67 %)	3 (4.35 %)	0.133
CRISPR1 + CRISPR3	0	0	0	–
CRISPR2 + CRISPR3	16 (11.11 %)	1 (1.33 %)	15 (21.74 %)	< 0.001
CRISPR1 + CRISPR2 + CRISPR3	0	0	0	–
CRISPR1 / CRISPR2	82 (56.94 %)	43 (57.33 %)	39 (56.52 %)	0.528
CRISPR1 / CRISPR3	42 (29.17 %)	13 (17.33 %)	29 (42.03 %)	0.001
CRISPR2 / CRISPR3	88 (61.11 %)	39 (52.00 %)	49 (71.01 %)	0.015
CRISPR1 / CRISPR2 / CRISPR3	92 (63.89 %)	43 (57.33 %)	49 (71.01 %)	0.062

p-value was calculated by one-tailed Fisher’s exact test and p-value < 0.05 was significant

Overall, the presence of CRISPR1 was found to have direct association with the absence of *tetM* and *ermB* (p = 0.043 and p = 0.032, respectively). The presence of CRISPR2 was directly associated to the absence of *vanA*, *aac6’-aph(2”)* and *aadE* genes (p = 0.0054, p = 0.049, and p = 0.015, respectively). As well as, the presence of CRISPR3 was directly associated to the absence of *tetM*, *ermB*, *aac6’-aph(2”)*, *aadE*, and *ant(6)* genes (p < 0.001 for all genes). In UTI isolates, relative association was found between the presence of CRISPR2 and the absence of *tetO* genes (p = 0.067, respectively). In DRC isolates, indirect association was found between the presence of CRISPR2 and the absence of *ermB*, *aac6’-aph(2”)*, *aadE* and *ant(6)* genes (p = 0.028, p = 0.006, p = 0.053, and p = 0.086, respectively), as well as, between presence of CRISPR3 and the absence of *aadE* gene (p = 0.021).

## Discussion

In this study, we examined 75 *E. faecalis* isolates from patients with UTIs and 69 *E. faecalis* isolates from patients with DRC infection to determine the relationship between CRISPR loci and antibiotic resistance among *E. faecalis* isolates. The occurrence of antibiotic resistance genes, the *cas* genes or resistance to antibiotics showed no distinct distribution within the groups of RAPD-PCR clustering, which are consistent with Linderstrauss et al. [27], who that found similar results for the occurrence of virulence genes and the *cas* genes. The current study demonstrated that the presence of CRISPR loci was variable among *E. faecalis* isolates, which the presence of CRISPR1 was in the lowest frequency among the isolates and CRISPR2 was the highest locus (53.47 %). In contrast our study, Palmer and Gilmore [12] found that CRISPR2 were present in all *E. faecalis* isolates. In addition, similar to our study, Palmer and Gilmore [12] found that one-third of isolates possess one of the CRISPR1 or CRISPR3. Our results demonstrated that co-occurrence of CRISPR1 and CRISPR3 was not found in the same isolates, which is similar to Palmer and Gilmore [12] and Linderstrauss et al. [27], while Burley and Sedgley [28] found the co-occurrence of these two loci in three endodontic isolates (0.03 %) out of a total of 88 endodontic, oral and hospital acquired isolates. Similarly to our study, Burley and Sedgley [28] demonstrated that the presence of CRISPR3 in endodontic isolates was more than CRISPR1 in comparison to UTI isolates. In addition, the presence of CRISPR loci among the DRC *E. faecalis* isolates was significantly higher than for UTIs isolates which were multi-drug resistant isolates, which as similar to Burley and Sedgley study [28]. The reason of higher presence of CRISPR loci among the DRC isolates is not clear but could confer low antibiotic resistance to them. In addition, Lyons et al. [29] suggested that the differences between incidences of CRISPR1 among the different species of enterococci may be associated with a tradeoff protection and adaptability, as well as, the differences in the habitats of different species of enterococci may be related to varying selective pressure exerted on them, which may results in a species-dependent distribution of CRISPR-*cas* systems. The possible mechanisms for these results are differential activity or expression of anti-CRISPR regulators or differential transcriptional regulation of *cas* genes in the conditions of in vivo and in vitro [30, 31] that may regulate CRISPR-*cas* systems of *E. faecalis* in different environments.

The lack of *cas* genes as functional genes among isolates with CRISPR2, as well as, the absence of some antibiotic resistance genes such as *vanA*, *aadE* and *aac6’-aph(2”)*, indicates that CRISPR2 alone does not confer immunity in *E. faecalis*. Similar results were observed by Palmer and Gilmore [12]. Hullahalli et al. [32] demonstrated that CRISPR2 could be reactivated in MDR strains for genome defense. Several studies demonstrated that the consensus repeat sequences of CRISPR1 and CRISPR2 loci are identical and they suggested that these two loci are functionally linked [17, 33–35]. Price et al. [35] demonstrated that an orphan CRISPR2 locus cannot provide defense on its own and requires CRISPR1-*cas* to provide genome defense against mobile genomic elements.

In our study, there was a significant association between phenotypic antibiotic resistance, the presence of antibiotic resistance-related genes and the absence of CRISPR loci. Similarly, Burley and Sedgley [28] and Palmer and Gilmore [12] reported that multi-drug resistance was associated with a lack of CRISPR loci. Similar to our study, they [28] found that the absence of antibiotic resistance was associated with the presence of CRISPR3, not CRISPR1. Several studies were proposed that the presence of CRISPR1 among *E. faecalis* is associated with the low prophage content in the strains such as *E. faecalis* OG1RF [17, 36, 37]. However, Bourgogne et al. [17] found that *E. faecalis* V583 lacks CRISPR1 and possesses seven prophage elements, which is in contrast with its function. They suggested that it might be associated with CRISPR1 locus variation in the *E. faecalis* species. These variably distribution was indicated in our study and study [12]. Palmer and Gilmore [12] demonstrated that the presence of CRISPR1 was present in 5/16 isolates and the presence occurred in variability between homologous of EF0672 and EF0673. In addition, their results indicated that the number of spacers in the CRISPR1 locus is varied and there is an unknown function gene between the 3’-end of CRISPR1 arrays and EF0673, which may confer variability of CRISPR activity in different species against antibiotic resistance genes [12].

Due to the fact that antibiotic resistance genes are commonly disseminated by plasmids in *E. faecalis* [38], CRISPR-*cas* may acts as a barrier to the acquisition of the antibiotic resistance genes. This is demonstrated by our results that the presence of CRISPR3 is significantly associated with the absence of some antibiotic resistance genes acquired by horizontal gene transfer such as aminoglycoside, tetracycline and erythromycin resistance-related genes, which are supported by Palmer and Gilmore [12], who found similar results in a collection of 48 *E. faecalis* strains. Price et al. [35] demonstrated that CRISPR3-*cas* is active for sequence-specific genome defense, which was observed in availability of CRISPR3-mutant of T11 that acquired *cas9* (Δca*s9* + CRISPR3) to interference and impacts on pAD1 acquisition. They also observed that deletion of only two loci can lead to a significant reduction in genome defense against clinically mobile genome elements [35].

This study supported that the occurrence of CRISPR loci is associated with the reduction of acquired antibiotic resistance genes, demonstrated with a reduced level of antibiotic resistance in *E. faecalis*. The inverse relationships between CRISPR loci and phenotypic and genotypic antibiotic resistance may provide novel insights to combat with the infection caused by resistant pathogens. CRISPR loci and other genetic markers could be used for infections control by *E. faecalis*, to give insights into their phenotypic traits and genetic contents, as well as, to differentiate low-risk strains of *E. faecalis* from high-risk strains.

## Supplementary Information

**Additional file 1: Table S1.** Primers used for the detection of antibiotic resistance genes and CRISPR-associated genes among *E. faecalis* isolates.

## Data Availability

All data and materials (DNA samples) are available by request from Author.
