# Supplementary material for: Role of CRISPR-Cas system on antibiotic resistance patterns of Enterococcus faecalis
Source: Ann Clin Microbiol Antimicrob. 2021 Jul 28;20:49. doi: 10.1186/s12941-021-00455-6 (PMC8317297; doi:10.1186/s12941-021-00455-6)
Supplement: Supplementary file 1 — Additional file 1: Table S1. Primers used for the detection of antibiotic resistance genes and CRISPR-associated genes among E. faecalis isolates. [file 12941_2021_455_MOESM1_ESM.docx]

**Additional file**

Table S1. Primers used for the detection of antibiotic resistance genes and CRISPR-associated genes among *E. faecalis* isolates.

| Primers name | Sequences | Amplified size (bp) | Reference |
| --- | --- | --- | --- |
| ddlE-F | ATCAAGTACAGTTAGTCTTTATTAG | 841 | [23] |
| ddlE-R | ACGATTCAAAGCTAACTGAATCAGT |  |  |
| tetM-F | GAACTCGAACAAGAGGAAAGC | 740 | [39] |
| tetM-R | ATGGAAGCCCAGAAAGGAT |  |  |
| tetO-F | AACTTAGGCATTCTGGCTCAC | 515 | [39] |
| tetO-R | TCCCACTGTTCCATATCGTCA |  |  |
| blaZ-F | ACTTCAACACCTGCTGCTTTC | 173 | [40] |
| blaZ-R | TGACCACTTTTATCAGCAACC |  |  |
| ermA-F | TATCTTATCGTTGAGAAGGGATT | 139 | [40] |
| ermA-R | CTACACTTGGCTTAGGATGAAA |  |  |
| ermB-F | CTATCTGATTGTTGAAGAAGGATT | 142 | [40] |
| ermB-R | GTTTACTCTTGGTTTAGGATGAAA |  |  |
| aadE-F | GCCCTTGGAAGAGTTAGATAATT | 198 | [41] |
| aadE-R | CGGCACAATCCTTTAATAACA |  |  |
| ant(6)-F | ACTGGCTTAATCAATTTGGG | 597 | [42] |
| ant(6)-R | GCCTTTCCGCCACCTCACCG |  |  |
| aac(6')-aph(2")-F | CCAAGAGCAATAAGGGCATA | 220 | [43] |
| aac(6')-aph(2")-R | CACTATCATAACCACTACCG |  |  |
| vanA-F | CATGAATAGAATAAAAGTTGCAATA | 1030 | [44] |
| vanA-R | CCCCTTTAACGCTAATACGATCAA |  |  |
| vanB-F | GTGACAAACCGGAGGCGAGGA | 433 | [44] |
| vanB-R | CCGCCATCCTCCTGCAAAAAA |  |  |
| CRISPR1-*cas csn1*-F | CAGAAGACTATCAGTTGGTG | 783 | [12] |
| CRISPR1-*cas csn1*-R | CCTTCTAAATCTTCTTCATAG |  |  |
| CRISPR1-*cas* loci-F | GCGATGTTAGCTGATACAAC | 315 | [12] |
| CRISPR1-*cas* loci-R | CGAATATGCCTGTGGTGAAA |  |  |
| CRISPR2 loci-F | CTGGCTCGCTGTTACAGCT | variable | [12] |
| CRISPR2 loci-R | GCCAATGTTACAATATCAAACA |  |  |
| CRISPR3-*cas csn1*-F | GCTGAATCTGTGAAGTTACTC | 258 | [12] |
| CRISPR3-*cas csn1*-R | CTGTTTTGTTCACCGTTGGAT |  |  |
| CRISPR3-*cas* loci-F | GATCACTAGGTTCAGTTATTTC | 224 | [12] |
| CRISPR3-*cas* loci-R | CATCGATTCATTATTCCTCCAA |  |  |
